# TrustNet: a lightweight network with integrated uncertainty quantification and quantitative explainable AI for ischemic stroke detection in CT images

**DOI:** 10.1038/s41598-026-37169-8

**Published:** 2026-02-19

**Authors:** Mahesh Anil Inamdar, Anjan Gudigar, U. Raghavendra, Aryaman Kaprekar, Massimo Salvi, Silvia Seoni, Girish R. Menon, Filippo Molinari, U. R. Acharya

**Affiliations:** 1 Manipal Institute of Technology, Manipal Academy of Higher Education, Manipal, 576104 India; 2 Biolab, PolitoBIOMed Lab, Department of Electronics and Telecommunications, Politecnico di Torino, Corso Duca degli Abruzzi 24, Turin, 10129 Italy; 3 Department of Neurosurgery, Kasturba Medical College, Manipal Academy of Higher Education, Manipal, 576104 India; 4 School of Mathematics, Physics, and Computing, University of Southern Queensland, Springfield, QLD 4300 Australia; 5 Centre for Health Research, University of Southern Queensland, Springfield, Australia

**Keywords:** Ischemic stroke detection, Deep learning, eXplainable AI, Uncertainty quantification, Monte Carlo dropout, Computational biology and bioinformatics, Engineering, Health care, Mathematics and computing, Medical research

## Abstract

Diagnosing ischemic stroke from computed tomography (CT) images is a highly challenging and detailed process that requires precise and careful analysis by a medical professional. Deep learning techniques offer an effective solution to this issue because of their remarkable performance. Nevertheless, most of those methods still lack the uncertainty quantification (UQ) and eXplainable artificial intelligence (XAI) features, which are essential for clinical practice and acceptance. We present TrustNet, a small but powerful convolutional neural network that uses Monte Carlo dropout and quantitative Grad-CAM. This technique helps visualize the issues related to two independent factors: uncertainty in the model’s classification and inconsistency in recognizing the relevant visual features. The model was validated on a set of 2023 brain CT scans and compared with networks that are generally used for classification purposes. TrustNet was able to achieve an accuracy of 94.67%, with 100% specificity, 91.6% sensitivity, and 100% precision, competing against various conventional architectures. The introduction of the UQ and XAI methods led to a consistent performance enhancement over the baseline models by limiting the number incorrect predictions, which is crucial for stroke diagnosis. With this performance, our approach can also provide an explanation for the reasoning and estimate confidence, which is essential for model deployment. This method is an indispensable tool for eliminating diagnostic bias and thus controlling the safety of AI in the clinical workflow.

## Introduction

Stroke is a major neurological disability caused when the cerebral blood supply is interrupted, leading to high rates of morbidity and mortality. Acute ischemic stroke, the second leading cause of death globally, remains a daunting challenge for healthcare^1,2^. The incidence of stroke varies according to geographic location; Western populations account for 10–15% of total intracranial atherosclerotic stenosis cases, and this number is as high as 46.6% in Asian populations^1,3^. Early detection with prognostication is essential to reduce triage^2^. CT is the primary imaging modality, preferred mostly because of its easy accessibility and speed; however, it is difficult to identify lesions because of their subtle intensity changes. Moreover, manual interpretation is time consuming and prone to observer-induced errors. The advent of Machine Learning (ML) and eXplainable Artificial Intelligence (XAI) in healthcare has made it possible to interpret imaging results in a way that supports health professionals in making informed evidence-based decisions^3–7^. In addition to accuracy in detecting strokes, special attention is given to individualized treatment approaches^8,9^. AI-powered operations are opaque and act as “black box” systems, which limits their clinical utility and trust across clinicians^10–12^. Hence, there is an urgent need to develop transparent AI diagnostic systems that can help interpret and identify patterns and make confident diagnoses.

### Explainability in stroke detection models

One of the most important aspects of AI decision-making for human beings is explainability, which, however, should not be confused with transparency. The latter refers to the extent to which the inner workings of the respective system can be seen and understood by a human being. In this context, the complexity of stroke detection is essentially in high demand for both speedy and accurate predictions, which ultimately leads to great advantages for patients^9,13,14^. As these models gain prominence, the need for clinicians to interpret AI-generated insights has become increasingly important^15^. Gradient-based visualization methods such as Grad-CAM and integrated gradients are designed to highlight affected areas^15^, thus fostering communication between AI and clinicians^16,17^. Here, it’s important to note the distinction between explainability and interpretability in AI models is as follows. Interpretability pertains to the extent to which a human can understand the mechanics and decision-making processes within the internal working of a model, whereas explainability attempts to explain the outputs of more black-box-like model interpreters post hoc^18,19^. For example, decision trees or linear regression models are inherently interpretable, as their input-to-output transformation process can be easily traced. Conversely, in the case of complicated models, such as deep neural networks, one usually needs other external interpretation methods, e.g., Local Interpretable Model-agnostic Explanation (LIME) and SHapley Additive exPlanations (SHAP)^1,20^. With all the promising applications of XAI in medical imaging, little has been explored in ischemic stroke detection. The few existing methods produce visual attention maps but do not attempt to measure, either technically or clinically, the correctness of the explanations or their contribution to boosting diagnostic confidence^10,21^. This points to an enormous gap in the literature, where XAI techniques for stroke detection are mostly qualitative and do not possess the proper rigour to prove their clinical utility^22^. To our knowledge, no established approach exists to systematically select the best XAI method for stroke detection or to extract meaningful quantitative results that can directly impact diagnostic performance.

### Uncertainty quantification in stroke detection

In stroke detection, UQ plays a major role, as it adds an extra layer of reliability and interpretability to each predictive model. These models not only provide better predictions but also enhance clinical decision-making through reliable model outputs. Generally, methods of UQ are broadly classified into aleatoric and epistemic uncertainties. Aleatoric uncertainty denotes uncertainty due to randomness in data, whereas epistemic uncertainty occurs due to imperfect knowledge or incomplete information concerning the system in question^23,24^. In medical imaging applications, a myriad of techniques, including Bayesian approximation and ensemble learning, have been employed to consider such uncertainties^10,25^. In clinical practice, it is important for practitioners to observe the role of uncertainty in clinical data while limiting its variability in the predicted output. Hemodynamic simulation could then rely on such a quantification because it is critical for patient-specific scenarios^9,26^. For example, incorporating UQ in clinical trials enhances outcome interpretability, facilitating clinical applications^27–29^. Moreover, UQ methods provide insight into prediction credibility, helping end-users understand both limitations and reliability of DL models^30^. Herzog et al.^30^ undertook the first work with uncertainty application in ischemic stroke analysis. In their approach, uncertainty was implemented in a CNN in the analysis of diffusion-weighted MR images to produce uncertainty estimates that correlated strongly with false predictions. More specifically, by showing that uncertainty measures such as the Monte Carlo Dropout (MCD) variance were able to remove uncertain cases, the classification accuracy improved from $\:93\%$ to $\:95\%$. Even with the promise of such gains, a great majority of UQ techniques in medical imaging have rarely been well calibrated^31^, and there is no standard uncertainty metric shared across diverse clinical contexts^32,33^.

### Objectives and contributions

While DL models can achieve high performance in ischemic stroke detection, their “black box” nature significantly limits their clinical adoption in acute care settings where transparency is crucial^34^. This research proposes a novel approach that combines UQ with XAI to address this challenge. Thus, AI-assisted stroke diagnosis, which is more trustworthy and interpretable for neurologists and radiologists, is needed. The key contributions of this work are as follows:

- *Proposed lightweight CNN architecture (TrustNet)*: TrustNet is a highly compact model with only 0.66 million parameters and is designed specifically for ischemic stroke detection.
- *UQ*: We use MCD^35^ for UQ, which allows the model to provide a confidence estimate for the prediction and to identify the uncertain instances for the expert to review. This is very important in the clinical workflow.
- *eXplainable AI (XAI) with a quantitative approach*: A preliminary intensity-based approach using Grad-CAM^36^ has been proposed to evaluate the consistency of model attention during stochastic inference. This is offered as an exploratory technique with UQ to reduce classification errors in situations where the model’s attention is spread or not dependable.
- *CT-based stroke classification model that integrates UQ with an XAI model*: To the best of our knowledge, this is the first instance in which a model for CT-based ischemic stroke classification has been constructed utilizing the UQ and XAI methods together in a single compact framework.

### Motivation

The process of stroke diagnosis by means of CT imaging is a critical but very difficult task for doctors. We provide a few rather basic reasons that have been the driving force of our research:

- *The usefulness of interpretability in clinics*: CT scans of strokes require that AI models produce not only accurate results but also interpretive results, which in turn help establish clinical trust and thereby provide support to decision-makers in their daily practice^1^.
- *The necessity of confidence estimation*: Without UQ, models can offer overconfident and possibly erroneous predictions, which is a very problematic situation, especially in critical applications such as stroke detection^30^.
- *Current models lack explainability*: Present-day DL models generally perform as “black boxes” and are devoid of any significant explainability tools, which renders them unfit for clinical deployment^34^.
- *Need for lightweight deployable models*: Many state-of-the-art networks are computationally heavy and impractical for real-time use in resource-constrained or bedside clinical settings^38,39^.

To bridge these gaps, we introduce TrustNet, a lightweight CNN that simultaneously integrates UQ (via MCD) and XAI (via Grad-CAM). Moreoever, our proposed methodology addresses Sustainable Development Goals 3, i.e., good health and well-being. The reminder of the paper is organized as follows. The initial sections provides a review of current state‑of‑the‑art techniques for ischemic stroke detection and presents a detailed overview of the proposed method. Further, the experimental results are outlined in the results section. Finally, an in‑depth discussion of the overall work, its implications for reliable stroke detection, and the conclusion of the present study are presented in the subsequent sections.

## Related works

There have been credible research studies in this domain in the recent past, with models enabling explainability and gauging confidence in prediction factors. To help the reader, we have collected and compiled studies published in the past 5 years (2020–2025) employing CT/MRI as the basic imaging modality.

### UQ-based studies

Authors Herzog et al.^30^ and Konathala et al.^37^ have employed Bayesian CNN to identify patients who have had ischemic stroke, providing uncertainty estimates alongside predictions to enhance reliability. Although there exists subtle difference in their approaches, Herzog et al. integrate Bayesian inference for MRI based patient level diagnosis, while Konathala et al. adopted variation Bayesian convolutional layers for uncertainty aware segmentation. Wang et al.^38^ proposed a distillation driven brain stroke region segmentation framework through multi-modal supervision with uncertainty modelling to refine the delination results using brain NCCT images. Molchanova et al.^14^ designed a lightweight custom CNN with three convolutional layers and heavy preprocessing to handle domain variation in CT images. Their internal and external dataset evaluation thus shows strong generalizability, with $\:97.2\%$ internal accuracies and $\:89.7\%$ external accuracies, indicating that the network can adapt well. However, their method provides strong predictive performance but does not incorporate explicit UQ into the model, thus leaving the issue of overconfident outputs unaddressed.

### XAI-based studies

Ozaltin et al.^39^ proposed a custom CNN architecture, referred to as OzNet, with mRMR feature selection followed by traditional classifiers such as SVM and naïve Bayes for stroke classification on CT images. While it achieves an accuracy of $\:98.42\%$ and an AUC value of $\:0.99$ across a public dataset, the method is far more concerned with feature selection. Although explainability is partially addressed through feature-ranking methods, no deep XAI visualization techniques (e.g., saliency maps) have been employed, limiting clinical interpretability. Wang et al.^40^ combined explainable ML with radiomics features (MRI), deep features and clinical data to classify cognitive disorders post stroke. Abdi et al.^41^ proposed a computationally efficient CNN optimized (hyper parameter tuning) pipeline for binary brain stroke classification. Their method enables visualization of decision relevant regions through model-agnostic explainability techniques. Gerbasi et al.^42^ used radiomic features from diffusion-weighted imaging and T2-FLAIR MRI during follow-up to predict outcomes in AIS patients. The use of SHAP for interpretability revealed key features contributing to prognosis, providing insight into lesion pathophysiology. Interpretable ML algorithms for poststroke neurological prognosis were developed by Wei et al.^43^, whereby SHAP was deployed to recognize the clinical variables and visualize their effects on detailed insight into model predictions. Nhlapho et al.^44^ designed a multimodal interpreted AI model that integrates radiomic features, DL features, and imaging features at several semantic levels. SHAP, Grad-CAM, and guided Grad-CAM were employed to enhance model explainability. Chagahi et al.^45^ employed methods such as Grad-CAM, Grad-CAM++, integrated gradients, and saliency mapping to provide visual explanations of model decisions. Gurmessa et al.^46^ used SHAP, LIME, and Vision Transformer (ViT) attention maps to explain the classification of stroke from CT scans, allowing for multilevel mobility and feature-based interpretability. Brändli et al.^47^ presented work to adapt Grad-CAM and occlusion to deep transformation models to highlight brain regions salient for predicting outcomes. It uses deep ensembles to obtain uncertainty-aware probability estimates to increase interpretability and trust in multimodal stroke analysis. Despite competitive performance, the lack of uncertainty quantification and interpretability limits the model’s clinical applicability. Kalyansundaram et al.^48^ integrated Grad-CAM to visualize salient regions within the segmented affected areas, enabling clinicians to validate the model’s spatial focus during classification. For uncertainty quantification, confusion entropy was introduced to identify predictions with low confidence, thus enhancing trust and enabling cautious interpretation of borderline cases. While these XAI techniques did not explicitly quantify uncertainty, they provide a foundation for assessing model confidence and supporting clinical trust.

### UQ + XAI predictions-based studies

Saeed et al.^49^ proposed a unique approach to segment brain affected regions and classifies their types using Bayesian optimized network. This network makes the decision transparent using Grad-CAM and reports prediction uncertainty with entropy computation. Inamdar et al.^51^ proposed a method to split CT images into patches to classify stroke cases using dual attention network with uncertainty and explainability modules. A summary of all the works is presented in Table 1.

**Table 1:** Summary of related works.

Researcher (et al.)	Methods	Dataset	Results
Herzog et al. (2020)^30^	Bayesian CNN + MCD	DW-MRI	Acc: $\:95.89\%$
Konathala et al. 2024^37^	U-Net	MRI (T2-FLAIR, BraTS 2020)	Whole tumor segmentation F1-score: 0.877, Mean IoU: 0.792
Wang et al. (2024)^38^	nnU-Net (teacher & student)	NCCT + DWI / CTP (multi-modal)	ISLES2018 dataset: Dice: 0.4841 ± 0.1801
Molchanova et al. (2025)^14^	Deep Ensembles + LSU	Basel: 163 Lausanne:43	DS: 0.617
Hossain et al. (2025)^10^	ViT + LSTM with SHAP XAI	CT (Rajshahi region)	Acc: $\:96.61\%$
Özaltın et al. (2022)^39^	Custom CNN (OzNet) + mRMR + SVM/NB	Kaggle database 1900 CT images	$\:\mathrm{A}\mathrm{c}\mathrm{c}:\:98.42\%,\:\mathrm{A}\mathrm{U}\mathrm{C}:0.99$
Wang et al. (2024)^40^	3D ResNet + Radiomics fusion	3D MRI	Acc: $\:92\%$
Abdi et al. (2025)^41^	Custom CNN (3 conv layers) + preprocessing	Public CT and external dataset	Val. Acc: $\:97.2\%;$ Prec/Sens: $\:96\%;$ External:$\:89.7\%$
Gerbasi et al. (2022)^42^	Radiomics + XGBoost	MR CLEAN–NO; 164 AIS patients	AUC 0.85 Acc: 79% , Prec: 78% .
Wei et al. (2024)^43^	Clinical-feature ML LASSO + SHAP	eICU‑CRD + MIMIC‑IV	Logistic Regression AUC: 0.887
Brändli et al. (2025)^47^	Deep Transform Model + Occlusion/Grad-CAM	Multimodal stroke data (407 patients)	AUC ≈ 0.81
Kalyanasundaram et al. (2024)^48^	Dual-stream net + Extra Trees + RFECV	Multimodal MRI + Clinical	Acc: 98%, F1 Score: 0.98
Saeed et al. (2024)^49^	DeepLabV3 + + Hybrid CNN with entropy	BraTS 2018/2020 ($\:285$ & $\:369$ subjects)	$\:\mathrm{A}\mathrm{c}\mathrm{c}:\:97\%,\:\mathrm{E}\mathrm{n}\mathrm{t}\mathrm{r}\mathrm{o}\mathrm{p}\mathrm{y}:0.26$
Inamdar et al. (2025)^50^	Dual attention + Adaptive RVFL	CT (multicenter, 7,842 images)	Acc: $\:92.42\%$
Inamdar et al. (2025)^51^	Dual attention (DAT + CAT) + patch embeddings + ensemble ML	2,023 CT images	Acc: 99.51%

Acc: Accuracy, Prec: Precision, Sens: Sensitivity, Spec: Specificity, AUC: Area Under Curve.

## Materials and methods

**Fig. 1:**
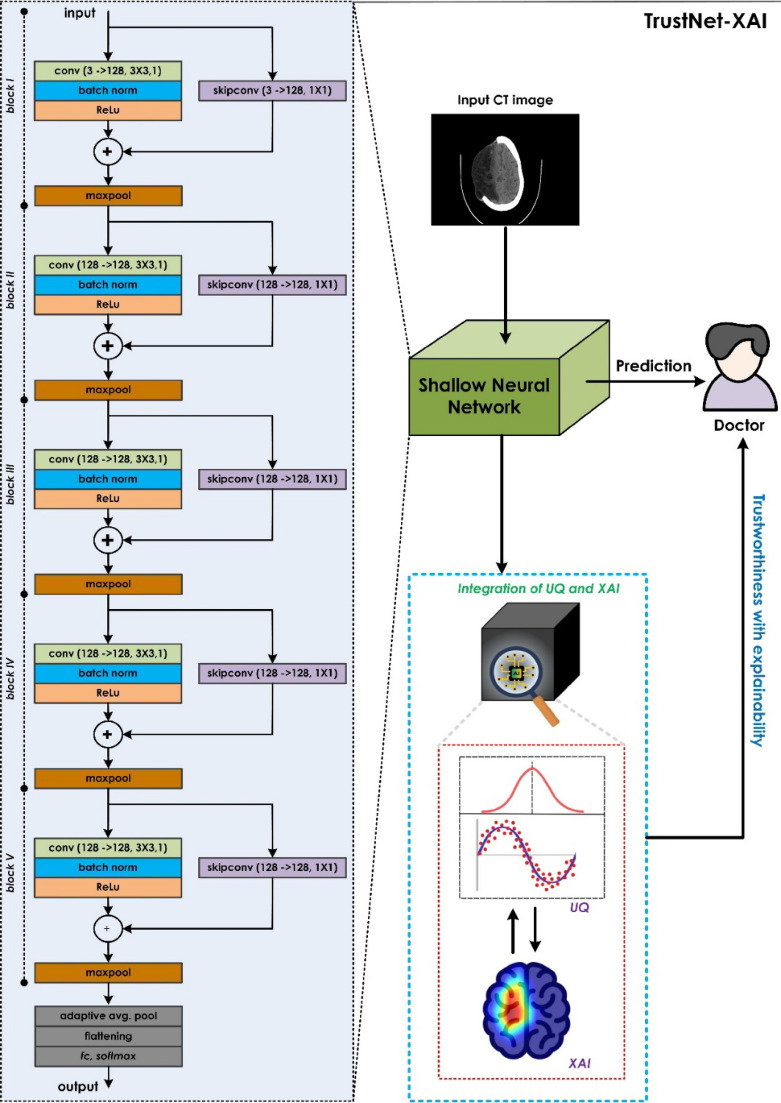
Overview of the proposed approach. The framework integrates a lightweight convolutional neural network optimized for ischemic stroke detection with two complementary components: an uncertainty quantification module based on MCD and an explainability module using Grad-CAM.

This study suggests integrating UQ with XAI to improve the clarity and dependability of AI-assisted stroke diagnosis for healthcare providers. Overall framework of the proposed method is illustrated in Fig. 1.

### Dataset

The study was conducted in accordance with the relevant ethical guidelines and regulations. The experimental protocol and data usage procedures were reviewed and approved by the Kasturba Medical College and Kasturba Hospital Institutional Ethics Committee (IEC), Manipal Academy of Higher Education, Manipal, India, Dated: 21 November 2024, IEC1: 423/2024. The present study involved retrospective analysis of anonymized brain CT images with no direct subject interactions, therefore the requirement for written informed consent was formally waived by the IEC.

The private dataset comprises 2023 CT scans (1013 healthy and 1010 ischemic stroke images) acquired at the Department of Neurosurgery, Kasturba Medical College (KMC), Manipal, India, between 2012 and 2018. Our dataset included 72 males and 22 females, with an average of 32 slices per case for acute stroke and 30 slices for both normal and chronic cases. The annotation was performed by a domain expert to ensure high-quality labelling. Unfortunately, metadata such as National Institutes of Health Stroke Scale (NIHSS)  scores or time-to-imaging from symptom onset are not available. The dataset is not publicly available due to patient confidentiality and ethical restrictions.

Nonrelevant CT slices outside the brain areas were excluded during preprocessing to maintain data quality and focus on anatomical regions. All images underwent identical preprocessing step of normalization to [0,1] range.

**Fig. 2:**
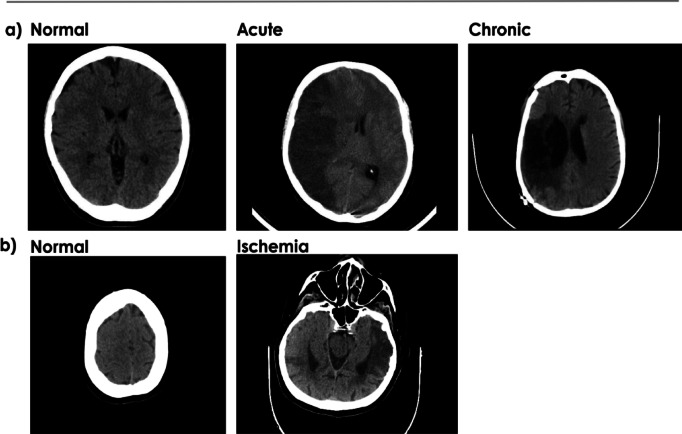
(**a**) Sample cases for the private database. (**b**) Sample cases for the Kaggle (public) database.

The dataset was split into 1458 training, 365 validation, and 200 test images (detailed in Table 2, with sample images shown in Fig. 2). The test set was fixed at 100 normal and 100 stroke cases for a balanced evaluation. All thresholds and hyperparameters were tuned exclusively using the validation set. For cross-validation, the remaining 1823 images were used in a 5-fold strategy, with Fold 5 selected for final evaluation due to its initially lowest performance. To test our model for the patient-wise image dataset, we performed the following split for training/validation/testing, with 30/20/16 and 22/2/4 patients for stroke and normal cases, respectively.

Additionally, to evaluate the generalization ability of our approach, we tested the trained model on a publicly available brain stroke dataset (available at link: https://www.kaggle.com/datasets/afridirahman/brain-stroke-ct-image-dataset accessed on 21 July 2025), which includes 1551 images of normal cases and 950 images of stroke cases.

**Table 2:** Dataset composition.

Subset	Slice-level			Patient-level		
	Healthy	Ischemic lesions	Total	Healthy	Stroke	Total
Train	731	727	1458	22	30	52
Validation	182	183	365	2	20	22
Test	100	100	200	4	16	20
Total	1013	1010	2023	28	66	94

### Proposed TrustNet architecture

Recently, CNNs have been extensively used for various medical applications, such as disease diagnosis and classification tasks^52–54^. The shallow but efficient CNN model named for brain stroke classification is shown in Fig. 1. There are five residual blocks arranged sequentially, with a $\:3\times\:3$ convolutional layer with $\:128$ output channels at the center. These layers handle finer spatial dependencies and local granular features that are related to lesion patterns. Batch normalization will then follow the convolution operation so that the internal covariate shift can be minimized, and the network will learn fastest with the greatest stability. On the other hand, ReLU activation is applied alone after this step to give the network nonlinearity to approximate more complicated decision boundaries. This output is then added with a projection of the block input (skip connection), at which point the channel dimensions are aligned before elementwise addition with the scanning path. Postresidual addition, dropout is applied to regularize learning via stochastically disabling activations, thus enhancing generalization on small and heterogeneous clinical datasets. The max pooling method follows the process of sampling the prominent activation patterns, and it is very important for the hierarchical abstraction of the model. After the fifth block, the network moves on to the lightweight classification head. The 2D adaptive average pooling layer reduces the spatial dimensions to a fixed output size, thus allowing flexibility in input resolution and parameter efficiency. The pooled feature maps are then flattened, and through a fully connected layer with linear activation, they are projected into a dense $\:128$-dimensional latent space where the raw activations are kept intact for either direct interpretability or downstream classification processes. This modularity ensures that each stage contributes to discriminative features being extracted by the convolutional units, contextual continuity being retained by the residual links, and the classification head consolidating evidence into this low-dimensional vector space, which is interpretable and ready to be used for decision making.

### Model calibration and uncertainty module

To strengthen the reliability of our ischemic stroke detection system, we integrated an uncertainty estimation framework based on Bayesian principles. MCD is commonly used to estimate epistemic uncertainty, which reflects uncertainty in the model parameters due to limited or imperfect training data. This form of uncertainty can be reduced with more data or better modelling. Conversely, aleatoric uncertainty is due to inherent noise or variability in the input data, such as low-resolution imaging or ambiguous features, which persists with the best training of an algorithm. Dropout prevents the trained model from just memorizing the training set by randomly dropping out the firing of a few neurons during the training phase. MCD extends the traditional dropout technique by applying it during both the training and testing phases, producing a predictive distribution rather than a single deterministic output.

**Fig. 3:**
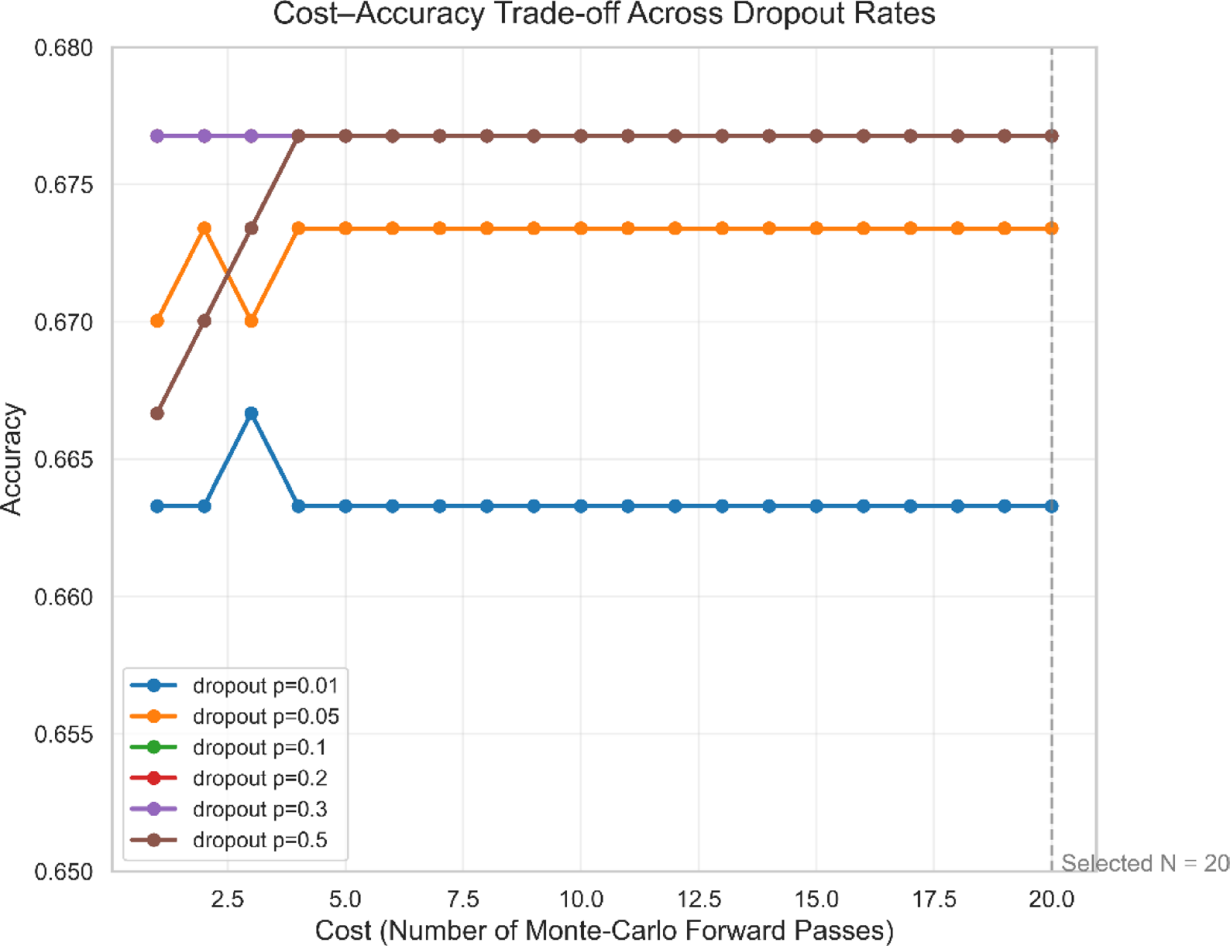
Cost-accuracy trade-off analysis.

Let M(θ*) represent our trained model, where *M* is the model architecture and θ* represents the weight parameters with dropout. To estimate uncertainty, we pass each unseen test sample through the model N times (stochastic forward passes), where *N* = 20, to obtain a posterior distribution^32,55^. The choice of *N* = 20 was determined empirically through a cost-accuracy trade-off analysis (Fig. 3). Our analysis shows that prediction accuracy increases with N but saturates around *N* = 10, with minimal improvements beyond this point despite increasing computational costs. Therefore, *N* = 20 provides a good balance between stable uncertainty estimates and computational efficiency, operating within the accuracy plateau region.

The class probability is calculated by averaging these stochastic predictions.

1 $$\:P(y|x)\:=\:\frac{1}{N}\sum\:_{n=1}^{N}P_{n}$$

where $\:P(y|x)\:$ denotes the output of the Softmax function (i.e., ŷ) for new data $\:x$, the label $\:y$, and the $\:n\:\in\:\:\{1,\:.\:.\:.\:,N\}$ forward pass. To quantify prediction uncertainty, we utilized information presented via theoretical principles by computing the normalized entropy^56^:

2 $$\:H=-\sum\:_{n}P(y|x)\:\mathrm{l}\mathrm{o}\mathrm{g}\:\ P(y|x)$$

**Fig. 4:**
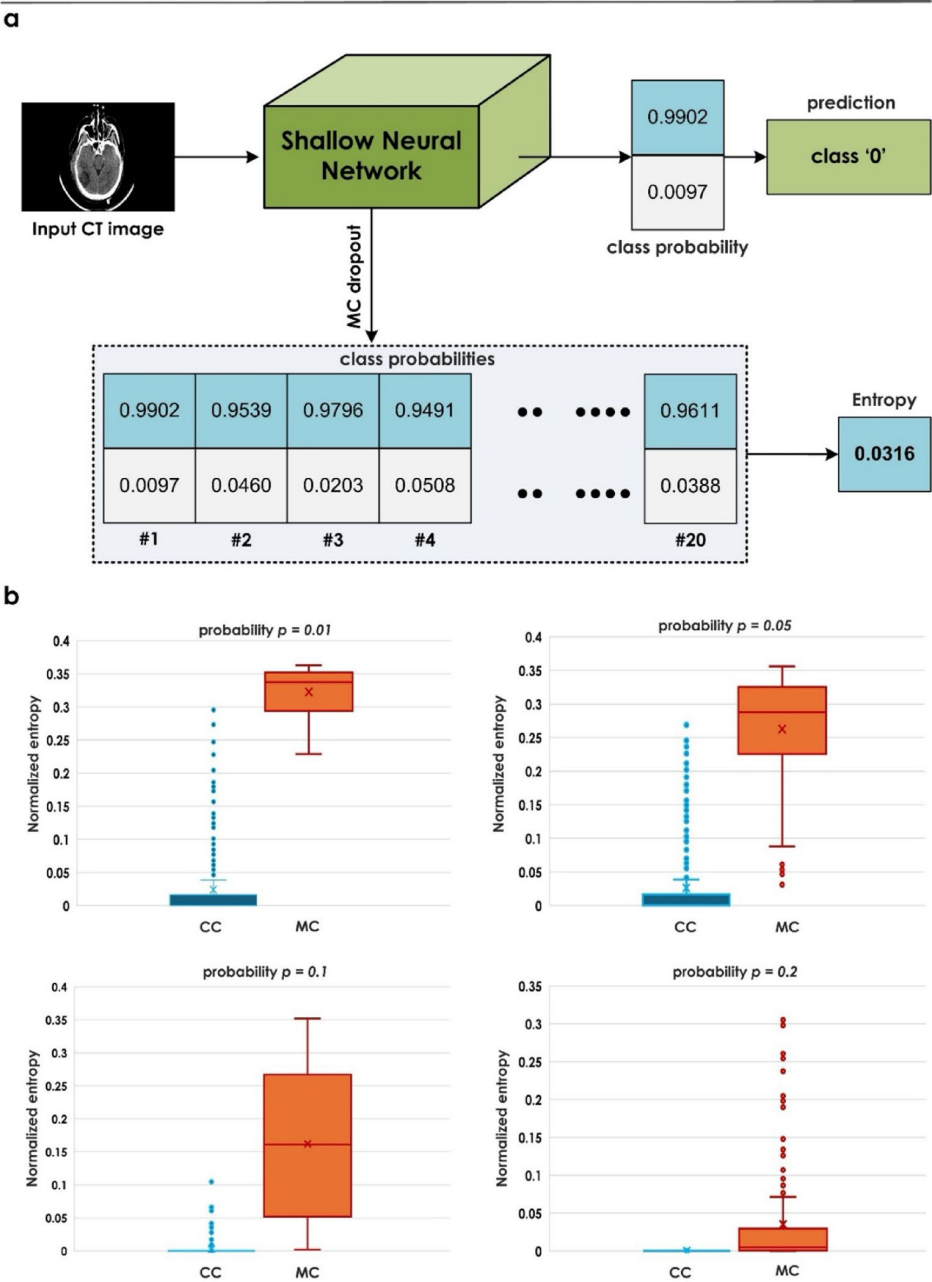
(**a**) MCD workflow for uncertainty estimation. (**b**) Boxplots showing the uncertainty for correctly vs. misclassified samples at different dropout rates.

The average entropy ranges between 0 (complete certainty) and 1 (complete uncertainty), providing an interpretable measure for clinicians. Figure 4a illustrates the entire MCD procedure, while Fig. 4b presents the uncertainty distribution across correctly classified (CC) and misclassified (MC) samples at different dropout rates $\:(p\:=\:0.01,\:p\:=\:0.05,\:p\:=\:0.1,\:p\:=\:0.2,\:p\:=\:0.3,\:p\:=\:0.5)$ in our validation dataset. Analysis of the boxplots revealed that a dropout rate of $\:p$ = 0.01 yielded the greatest separation between uncertainty distributions of correct and incorrect predictions, making it optimal for the inference step on the test set.

Based on this finding, we established decision boundaries for three dataset configurations: slice-wise split of private data, image-wise split of public data and patient-wise split of private data. This approach optimizes the sensitivity-specificity trade-off in uncertainty detection, minimizing both misclassifications and false positives.

During inference, each CT scan undergoes the same process: using a dropout probability of $\:p$ = 0.01, we generate an ensemble of predictions through multiple forward passes to calculate the entropy-based uncertainty metric (H). We classify predictions as reliable when H ≤ α, where α is the mean entropy. Cases where H > α are flagged for expert review and excluded from automated classification, helping prevent undetected misclassifications.

### Quantitative XAI and explainability module

For the assessment of model interpretability in uncertain situations, we applied a quantitative explainability framework that utilized several gradient-based visualization methods: Grad-CAM, Grad-CAM++, and Score-CAM. After comparative analysis of these methods, Grad-CAM was selected as our primary visualization technique due to its superior performance in obtaining localized activation patterns and lower Area Under Perturbation Curve (AUPC) values.

A total of two inference modes were used for every sample tested: the first mode was regular deterministic inference (dropout layers were shut off), whereas the second mode was MCD-based inference, in which stochasticity was introduced by turning on dropout layers during the inference (testing) phase. The dropout percentage during testing was equal to the most effective percentage found in the previous model calibration process (i.e. $\:p$=0.01).

Initially, following established literature, we evaluated the Pearson Correlation Coefficient (PCC) to measure consistency between saliency maps generated from both standard deterministic inference and MCD-based inference. However, we found that PCC values showed minimal variation across all explainability techniques, remaining consistently high. This uniformity was attributed to the inherent anatomical homogeneity of CT brain images, where similar structural patterns produced consistent saliency maps regardless of dropout-induced variations. Given PCC’s limited discriminative power, we developed an alternative approach based on Mean Saliency Intensity (MSI), as detailed in the following section.

#### Mean saliency intensity (MSI)

**Fig. 5:**
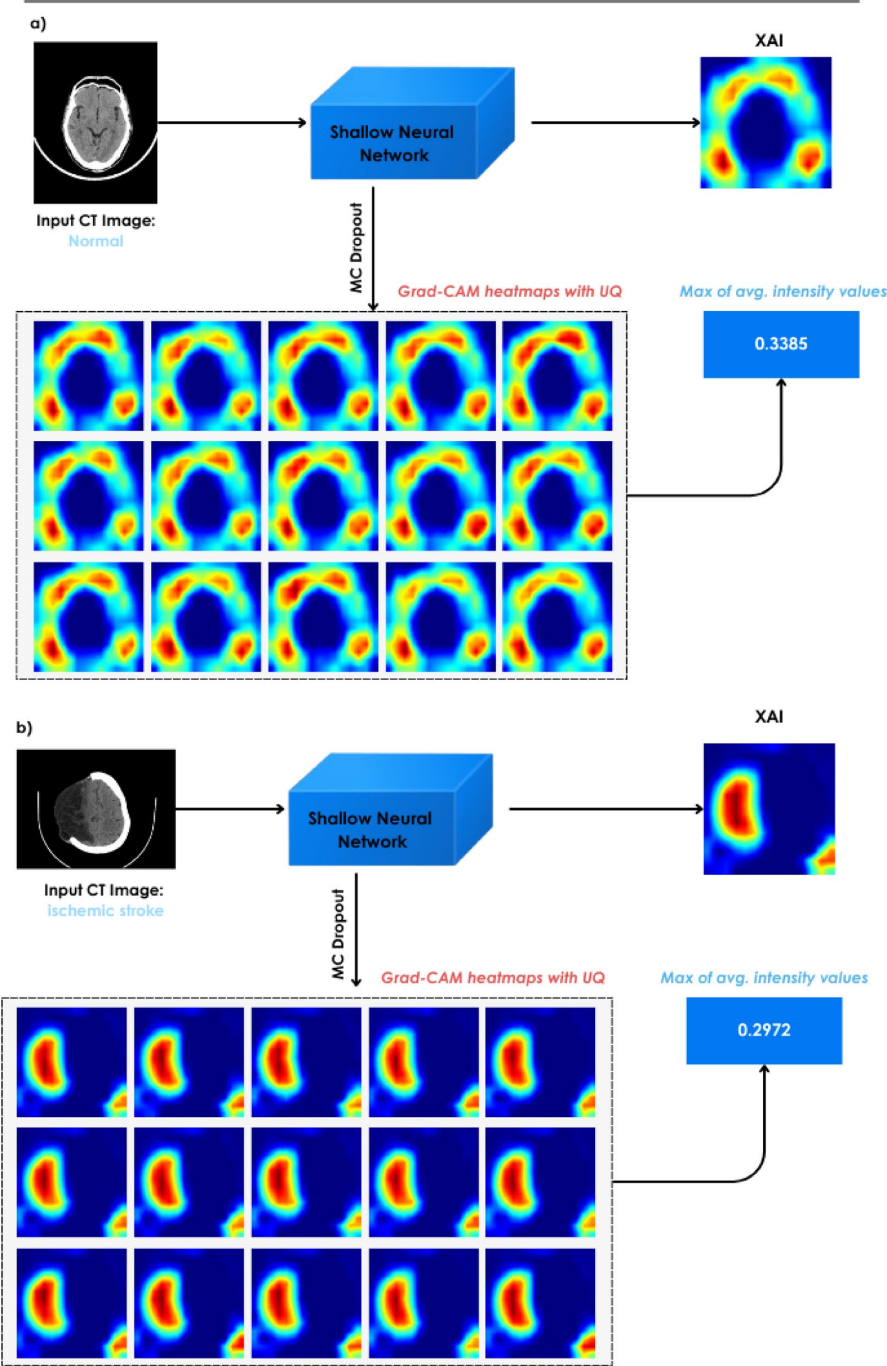
Illustrates the Grad-CAM heatmaps, with (**a**) representing a normal case and (**b**) representing an ischemia case.

For a given CT image $\:x$, let $\:C\left(x\right)$ be the saliency heatmap generated by explainability methods. This value is normalized in the range of [0–1] to make equal scaled comparisons across methods. The MSI for an image$x$ is defined as:

3 $$\:MSI\left(x\right)=\frac{1}{U.V}\sum\:_{i=1}^{U}\sum\:_{j=1}^{V}{C}_{i,j}\left(x\right)$$

where U and V are the height and width of the heatmap, respectively. This metric provided a more sensitive measure of the model’s confidence in highlighting relevant regions, as illustrated in Fig. 5 (Grad-CAM heatmaps for normal case 5a and ischemia case 5b). For each sample, we compare the MSI values obtained from both deterministic saliency maps and MCD-based maps. Our empirical analysis revealed that high MSI values indicated indiscriminate feature identification across the image, correlating with misclassification. In contrast, samples being correctly classified showed attention patterns that were more focused on well-defined regions with lower-to-moderate average intensity values. This means that the ability of the model to focus on the most relevant anatomical areas, as opposed to mere high-intensity activation across the image, is an indicator of successful classification.

Based on these observations, we developed a thresholding mechanism using the MSI values from all cases True Positive (TP), True Negative (TN), False Positive (FP) and False Negative (FN). For misclassified cases (FP and FN), we apply the following thresholding criteria:

4 $$\:D=min(\mathrm{max:}\left(MS{I}_{TP}\right),\:\mathrm{max:}\left(MS{I}_{TN}\right))$$

where $\:MS{I}_{TP}\:$ and $\:MS{I}_{TN}\:$are the average intensity values for the TP and TN cases, respectively. We then reverse the model’s initial classification for samples with $\:MSI\:\ge\:\:D$, whereas for samples with $\:MSI\:\le\:\:D$, we keep the model’s initial predictions. This rule essentially helps the model refine misclassified predictions with sufficient intensity in heatmaps. This intensity-based metric complements the entropy-based uncertainty quantification by identifying cases where the model shows high confidence but fails to properly localize discriminative features. The complete implementation is detailed in Algorithm 1.

### Performance metrics

The performance of the proposed model was evaluated using multiple classification metrics to assess its diagnostic accuracy. Accuracy provides the overall number of classes correctly allocated from among all samples^57^. Once projected into the information retrieval literature, precision and recall quantify the ability of the model to find real stroke cases (sensitivity) while minimizing its false alarms (precision)^58^. The F1 score was then computed as the harmonic means between precision and sensitivity to give an equated measure of performance. Conversely, specificity measures the true negative rate, i.e., how well the model refrains from incorrectly labelling nonstroke samples as stroke samples, a point of utmost relevance during clinical screening, which could otherwise culminate in unwarranted interventions and mismanagement of scarce resources. The AUROC was also calculated to ascertain the overall model class differentiation independent of threshold choice, in which the goal should be 1.0, indicating perfect classification. These metrics, together, constitute a complete model evaluation in ways orthogonal to each other and clinically pertinent in terms of diagnostic conduct.

**Algorithm 1:**
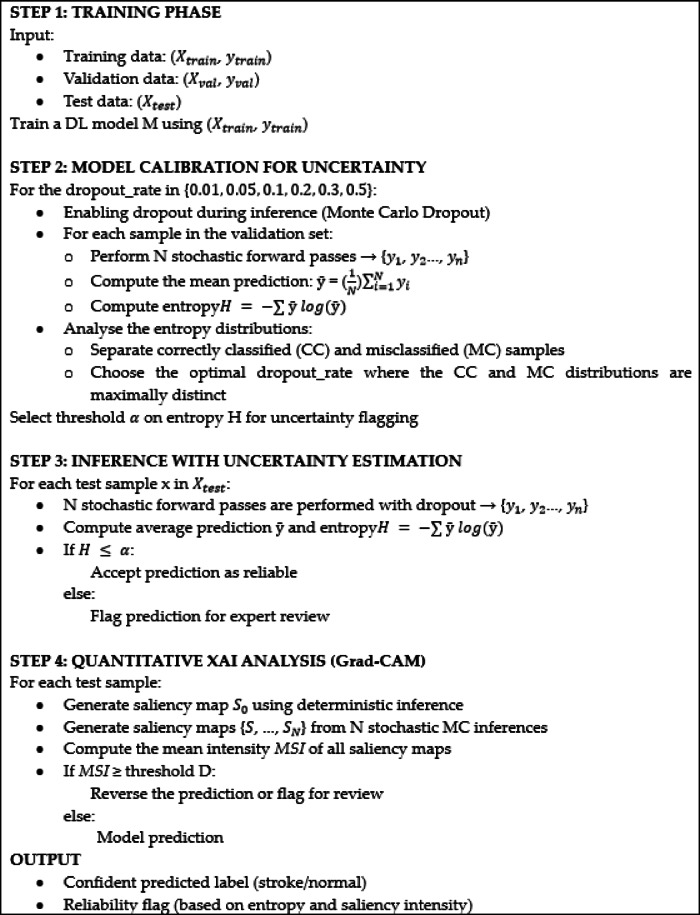
TrustNet+UQ+XAI.

## Results

The results obtained are presented here for each case: the baseline model, the model with UQ, and the model including UQ and XAI.

### Baseline model performance

Table 3 presents an overview of the performance of the baseline model across the training, validation, and test sets. In particular, the model achieves high accuracy over all phases: 99.71%, 99.42%, and 88.55% for training, validation, and test data, respectively. This shows that the model generalizes well and does not overfit considerably. The model effectively differentiates between stroke and non-stroke categories even when tested on varying data distributions. In terms of diagnostic accuracy, the model had a sensitivity or recall that was always very close to 100% at the minimum across all the sets, which means that the model never overlooked a true positive case, which is essential in clinical stroke detection, to avoid missed diagnoses. The model’s specificity, which measures the ability to correctly identify non-stroke cases, increases from 99.16% in training to 100% in both validation and test sets, demonstrating excellent performance in avoiding false positives. This is further reflected in the precision $\:\left(100\%\right)$ and F1 score $\:\left(89.12\%\right)$ on the test set, which continues to indicate a strong balance in performance even under stricter generalization.

**Table 3:** Baseline TrustNet results.

Set	Acc. (%)	Sens. (%)	Spec. (%)	Prec. (%)	F1 score (%)
Train	99.71	100	99.16	100	100
Validation	99.42	98.35	100	99.12	98.73
Test	88.55	83.08	100	100	90.76

### Performance of the proposed XAI + UQ approach

This section evaluates the performance of the proposed XAI + UQ framework for binary classification of ischemic stroke (class 0) versus normal cases (class 1), under both patient-wise and image-wise dataset split configurations. The framework follows a cascaded filtering strategy: first, an UQ module based on MCD (*N* = 20) filters out low-confidence predictions; subsequently, an XAI module analyses the retained predictions using Grad-CAM intensity values.

Table 4 summarizes the quantitative performance of the baseline, UQ-enhanced, and UQ + XAI models across private and public datasets, reporting accuracy, sensitivity, specificity, and precision with 95% Wilson confidence intervals. Across all evaluated settings, the integration of UQ consistently improves performance by reducing both FN and FP. The addition of XAI further refines the predictions, leading to more robust and interpretable decision-making. These improvements are particularly critical in stroke screening, where missed detections may result in severe delays in treatment.

**Table 4:** Performance with confidence interval (95% Wilson) of baseline, UQ, and UQ + XAI models across private (image-wise and patient-wise) and public datasets.

Dataset / model	Acc. (%)	Sen. (%)	Spec. (%)	Prec. (%)
Private dataset (patient-wise split)				
Baseline	88.5 [84.43, 91.69]	83.08 [77.29, 87.64]	100 [96.15, 100]	100 [97.75, 100]
Baseline + UQ	94.67 [91.26, 96.80]	91.6 [86.42, 94.94]	100 [96.15, 100]	100 [97.55, 100]
Baseline + UQ + XAI	94.67 [91.26, 96.80]	91.6 [86.42, 94.94]	100 [96.15, 100]	100 [97.55, 100]
Private dataset (image-wise split)				
Baseline	96.5 [92.95, 98.29]	93 [86.25, 96.57]	100 [96.30, 100]	100 [96.03, 100]
Baseline + UQ	98.41 [95.44, 99.46]	96.63 [90.55, 98.85]	100 [96.30, 100]	100 [95.72, 100]
Baseline + UQ + XAI	98.94 [96.22, 99.71]	97.75 [92.17, 99.38]	100 [96.30, 100]	100 [95.77, 100]
Public dataset (image-wise split)				
Baseline	93.5 [90, 96.5]	99 [96.7, 100]	88 [81.4, 93.9]	89.2 [83.2, 94.6]
Baseline + UQ	97.09 [94.19, 99.42]	100 [100, 100]	93.2 [86.76, 98.55]	95.2 [90.65, 99.02]
Baseline + UQ + XAI	98.3 [97.09, 100]	100 [100, 100]	95.89 [92.96, 100]	97.01 [94.90, 100]

**Fig. 6:**
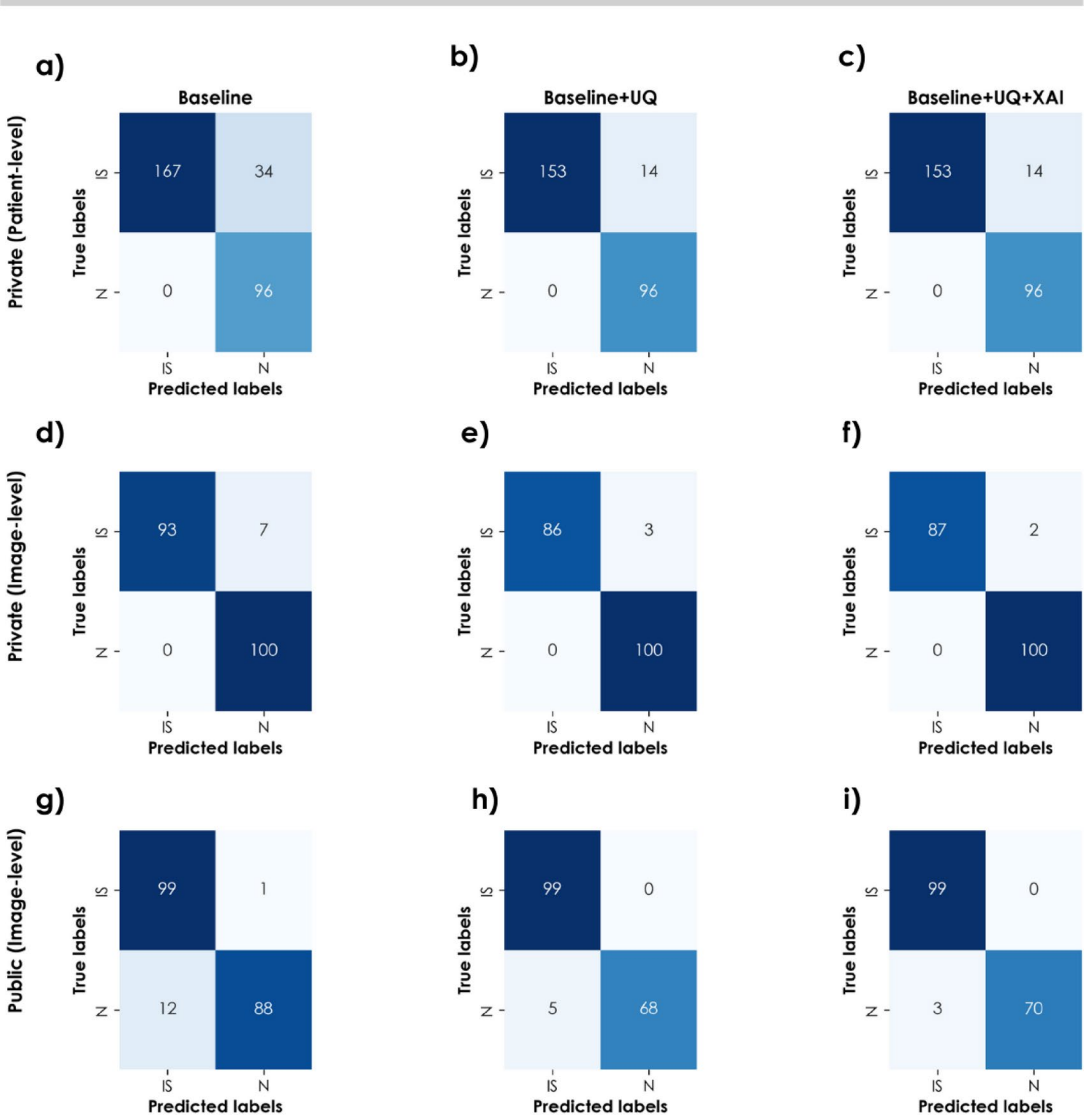
Confusion matrices for various test sets on the private and public datasets; IS: Ischemic stroke; N: Normal.

The qualitative impact of these improvements is illustrated through the confusion matrices shown in Fig. 6, which provide a detailed breakdown of classification errors for each experimental configuration.

For the private dataset (patient-wise split):

- In the baseline model (Fig. 6a), 34 FNs occur, reducing its sensitivity to approximately$\:\:83.08\%.$.
- After the UQ analysis (Fig. 6b), $\:34$ low-confidence predictions using Eq. 2 are removed, thereby reducing the FN to $\:14$ ($\:91.6\%$ sensitivity).
- After XAI analysis (Fig. 6c), as none of misclassified cases satisfy the criteria as mentioned in Eq. 4, values remain same.

The progressive refinement in predictive confidence, from baseline to UQ and finally XAI-enhanced outputs, suggests that integrating epistemic uncertainty estimation helps suppress low-certainty predictions, whereas XAI facilitates more interpretable and robust decision-making. The combination of both methodologies is indeed useful, as it facilitates the recognition of difficult cases from two different but complementary angles: (1) the uncertainty of the model regarding classification and (2) the reliability of the relevant visual features being identified. To further grasp these findings, we analysed the failure cases meticulously.

For the private dataset (image-wise split),

- In the baseline model (Fig. 6d), $\:7$ FNs occur, yielding sensitivity of approximately $\:93\%$ and an accuracy of $\:96.5\%$.
- After the UQ analysis (Fig. 6e), $\:11$ uncertain predictions are removed with Eq. 2, reducing the considered cases to $\:86$ and reducing the FN to 3, improving the sensitivity to $\:96.63\%$.
- After XAI analysis (Fig. 6f), a prediction is reversed based on the Eq. 4 values, improving the specificity to$\:\:97.75\%.$.

For the public dataset, where patient-wise splits are not available, the baseline model achieves an accuracy of 93.50% (Fig. 6g). Incorporating UQ improves accuracy to 97.09%, primarily driven by an increase in precision from 89.2 to 95.2% (Fig. 6h), indicating a substantial reduction in FPs. The addition of XAI further improves accuracy and precision to 98.3% and 97.01% (Fig. 6i), respectively, highlighting the complementary benefits of uncertainty estimation and explainability.

### Comparative analysis

The comparative analysis presented in Table 5 demonstrates the effectiveness of the proposed shallow architecture against a diverse set of classical convolutional encoders, ranging from well-established classification networks to lightweight architectures with respect to performance metrics and computational complexity. With an accuracy of $\:98.94\%,$ $\:97.75\%\:$sensitivity and an F1 score of $\:98.86\%$ for private dataset image level and accuracy of $\:94.67\%,$ $\:91.61\%\:$sensitivity and an F1 score of $\:95.62\%$ for private dataset patient level, TrustNet guarantees extremely reliable stroke classification. Keeping the number of parameters to a bare minimum of $\:0.66$ million and the byte size to a mere $\:2.54$ MB, it, in effect, is orders of magnitude lighter than heavyweight architectures such as VGG-16 ($\:134.27$ million parameters) and ResNet-$\:152$ ($\:58.15$ million parameters), which produce far inferior accuracies of $\:89\%$ and $\:91.5\%$, respectively. From a precision-sensitivity perspective, many deeper networks, such as ResNet-101 and ResNet-50, suffer from modest F1 scores ($\:71.17\%$ and $\:85.84\%,$, respectively), despite achieving $\:100\%$ sensitivity, highlighting a severe class imbalance handling issue and a propensity to overfit. On the other hand, networks such as EfficientNet-B0 and InceptionNet V3 achieve perfect scores across all the metrics but still require sufficient memory, which is significantly greater than that of our proposed model. MobileNetV2, while compact at $\:2.23$ million parameters, underperforms in sensitivity ($\:70\%$), suggesting that it misses critical stroke-positive cases, which is a dangerous shortcoming in clinical applications. Models such as ResNet 152 and Inception, although they yield the best results, are observed to make non-confident predictions and are a possible case of overfitting. The proposed network therefore strikes a unique and desirable balance between performance, interpretability, and deployment ability, making it particularly suitable for resource-constrained, real-time diagnostic systems, such as mobile health platforms and edge-enabled radiology tools.

**Table 5:** Comparison with classical classification networks.

Model	Acc (%)	Prec (%)	Sens (%)	Spec (%)	F1 score (%)	Param. size (Million)	No. of layers	GFLOPs	Inference time (ms)
ResNet 18^59^	90.5	98.8	82	99	89.62	11.18	21	3.65	3.936
ResNet 34^59^	98	96.15	100	96	98.04	21.29	37	7.36	5.284
ResNet 50^59^	83.5	75.19	100	67	85.84	23.51	54	8.26	8.533
ResNet 101^59^	59.5	55.25	100	19	71.17	42.5	105	15.73	15.700
ResNet 152^59^	91.5	100	83	100	90.71	58.15	156	23.2	23.016
InceptionNet V3^60^	100	100	100	100	100	21.79	98	5.71	16.126
MobileNet V2^61^	85	100	70	100	82.35	2.23	53	0.65	8.709
DenseNet 121^62^	99	98.04	100	0.98	99.01	6.96	121	5.79	21.236
EfficientNet B0^63^	100	100	100	100	100	4.01	82	0.83	12.783
VGG 16^64^	89	83.05	98	80	89.91	134.27	16	30.93	11.497
AlexNet^65^	84.5	76.34	100	69	86.58	57.01	8	1.42	1.759
**Proposed network**	**98.94**	**100**	**97.75**	**100**	**98.86**	**0.66**	**11**	**5.91**	**3.659**

The analysis presented in Table 6 further highlights the advantages of our proposed work over very recent human brain stroke classification studies and neurological disorder classification applications^15,66,67^. While some research projects excel in at least one of these dimensions—performance, uncertainty quantification, or explainability—most do not simultaneously address all three. For example, the Bayesian CNN proposed in^30,37^ achieves a high accuracy of 95.89% using MCD-based uncertainty estimation but lacks any explainability mechanism, thereby limiting clinical transparency. Similarly, the 3D ResNet and radiomic fusion strategy in^35^ introduces SHAP-based feature attribution but stops short of integrating any UQ framework and operates at a lower overall accuracy of $\:92\%$. Even advanced segmentation classification pipelines such as^14,38,39^, despite best lesion delination, typically trade off interpretability, depth, or computational compactness. A dual attention mechanism framework combined with random vector functional link was proposed in^50^, which was utilized for multicenter ischemic stroke detection and resulted in a remarkable 92.42% accuracy. The study stressed not only the significance of the attention mechanisms but also the cost-effective classification head. TrustNet, instead, is a shallow and fast model that incorporates both XAI (Grad-CAM) and UQ (MCD + saliency consistency analysis) in a single streamlined architecture. The proposed method not only outperforms or stands at par with deeper multimodal systems ($\:94.67\%$ accuracy, $\:100\%$ precision, $\:91.6\%$ sensitivity, and $\:100\%$ specificity) but also provides interpretability of outputs and strong confidence estimates. Our model also demonstrated strong performance on the public dataset, as reported in Table 6. This balanced convergence of performance, transparency, and uncertainty reasoning positions our model as a leading contribution to clinically deployable AI for ischemic stroke detection.

**Table 6:** Comparison with existing works in terms of objectives, strengths, limitations and presence of UQ / XAI tool.

Studies	Key objectives	Strengths	Limitations	UQ	XAI
Hossain et al. (2025)^10^	Stroke detection from CT	Powerful transformer backbone with interpretability	No UQ; interpretability results lack quantitative evaluation	✗	✓
Molchanova et al. (2025)^14^	MS lesion segmentation and uncertainty explanation	Novel Lesion Structural Uncertainty (LSU) metric	Lacks quantitative classification results	✓	✗
Herzog et al. (2020)^30^	Classify ischemic stroke lesions in DW-MRI	Early use of UQ; uncertainty guided misclassification handling	No XAI; modality limited to MRI	✓	✗
Özaltın et al. (2022)^39^	Stroke classification	Compact model with feature selection	No UQ; no XAI integration	✗	✗
Wang et al. (2024)^39^	Motor & cognitive disorder level classification	Combined deep & clinical features; interpretable SHAP-based output	No UQ; moderate accuracy	✗	✓
Saeed et al. (2024)^49^	Brain tumor segmentation and classification	UQ + XAI; segmentation & classification unified	Limited generalizability; MRI only	✓	✓
Abdi et al. (2025)^41^	Stroke classification from CT	Tested on external dataset; good generalization	No UQ; limited interpretability	✗	✓
Gerbasi et al. (2022)^42^	Classification of long-term functional outcome after acute ischemic stroke	Multicenter study with lesion texture heterogeneity	Implicit UQ	✗	✓
Wei et al. (2024)^43^	Classification of neurological outcome at ICU discharge	Large multicenter ICU cohorts	No Uncertainty Quantification	✗	✓
Brändli et al. (2025)^47^	Stroke outcome prediction using multimodal data	Multimodal attention visualizations	No UQ; outcomes not quantified	✗	✓
Inamdar et al. (2025)^50^	Multicenter ischemic stroke classification	Lightweight, fast; multi-institutional	XAI modules only	✗	✓
Inamdar et al. (2025)^51^	Ischemic stroke classification	Patch-level interpretability	Single center dataset	✓	✓
**Proposed work**	**Lightweight and interpretable ischemic stroke classification**	**TrustNet with integrated UQ (MCD) + XAI (Grad-CAM)**	**Private (patient and image wise)**,** Public**	**✓**	**✓**

### Ablation study

The ablation study presented in Table 7 evaluates the contributions of individual architectural components within the proposed TrustNet model by incrementally enabling or modifying blocks and convolution configurations. The key insights obtained are as follows. An extremely important step in hierarchical feature extraction is the steep jump in performance, from using only block 1 (81% accuracy) to both block 1 and block 2 (88.4% accuracy). Since the addition of a second residual block increases the specificity and precision, it is better at discriminating nonstroke cases; thus, it has fewer FP. The custom configuration (full TrustNet) achieves the best overall balance (93.5% accuracy, 89.2% precision, 99% sensitivity). This confirms that the proposed residual block design and feature depth are well tuned for stroke classification, preserving sensitivity while sharply enhancing model confidence in negative cases. This indicates that while receptive field diversity is slightly increased, it is also inhibited by introducing more redundant or less discriminative features for this binary task. Custom: The 64,128,256 model with deeper channels has a decrease in accuracy and sensitivity to 90.2% and 97%, respectively. Thus, it is probably overfitting or undergeneralizing due to feature oversaturation on a rather small dataset.

**Table 7:** Result of ablation study using various configurations of public dataset.

Various configuration	Acc. (%)	Prec. (%)	Sens. (%)	Spec. (%)	F1 score (%)
Block 1	81.0	73.1	98.0	64.0	83.8
Block 1, 2	88.4	86.5	96.2	82.1	91
Custom: 64,128,256	90.2	88.9	97.0	84.0	92.7
Custom: 64,128,256–3,5	91.3	88.7	97.5	85.5	92.8
Custom: 64,128–3,5	92.6	89.0	98.2	86.5	93.3
**Custom (proposed)**	**93.5**	**89.2**	**99.0**	**88.0**	**93.83**

### Perturbation-based validation of explanations

**Fig. 7:**
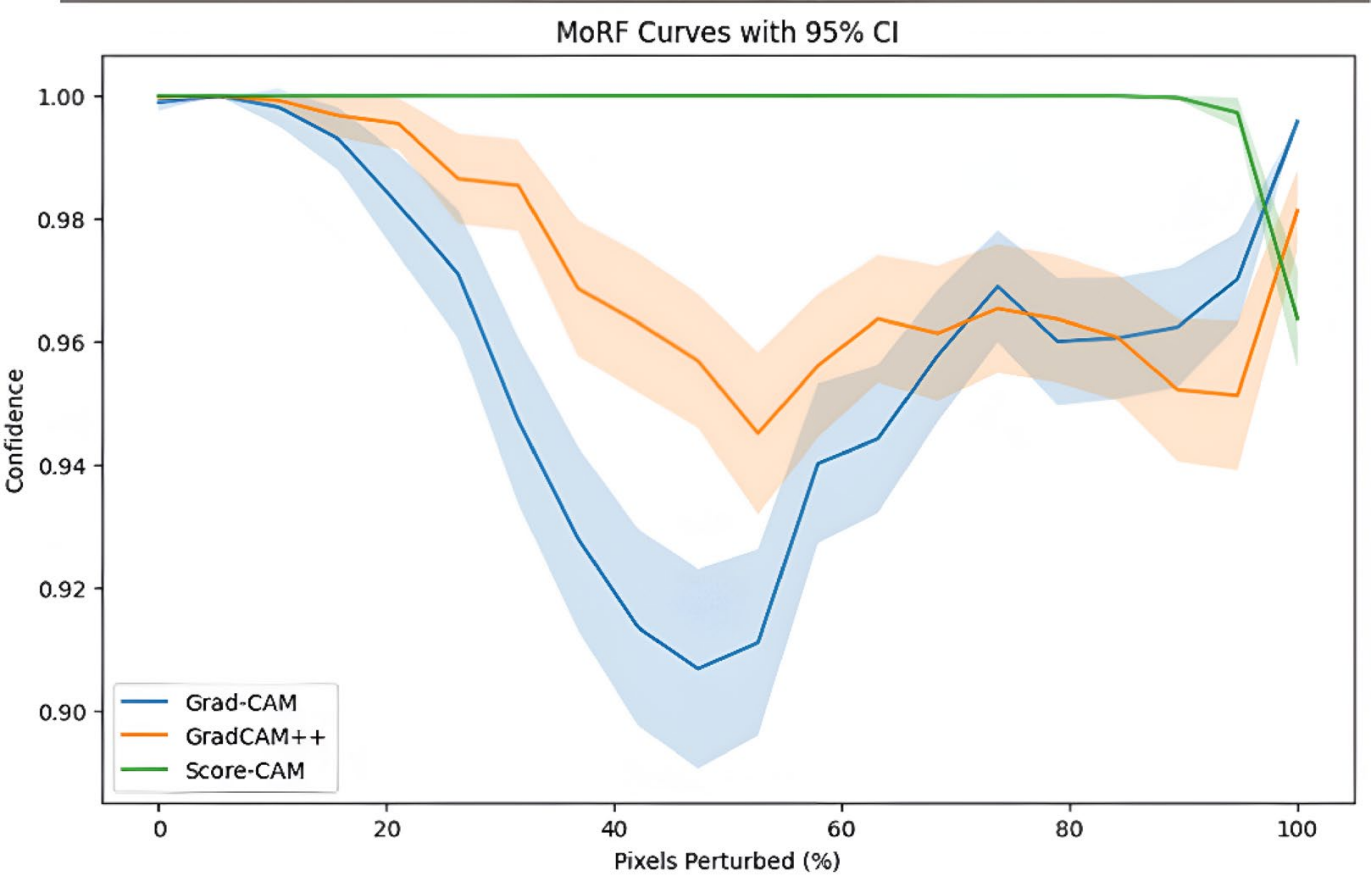
MoRF perturbation with confidence bands, for Grad-CAM, Grad-CAM++, and Score-CAM techniques.

Most relevant first (MoRF) perturbation curve is used to evaluate the performance of XAI techniques^68^. This technique involves removing important (identified) features and monitoring the impact on the yielded performance. This provides an idea of whether the metric truly captures regions important for the model’s decision. The authors of^69^ highlighted the MoRF as a robust and interpretable benchmark for assessing the alignment between model predictions and the explanatory relevance assigned by attribution methods. In general, a lower MoRF AUC indicates greater faithfulness, as model performance decreases more quickly when its most relevant regions are removed. Figure 7 shows the confidence vs. pixel perturbed for all three approaches with confidence interval of 95%. Both showcase that Grad-CAM shows credible sensitivity towards loss of pixels, making it a viable approach to generate saliency maps.

To further strengthen the analysis, we evaluated perturbation curves with mean confidence trajectories (including confidence bands) and AUPC, as shown in Table 8. The MoRF curves demonstrate that Grad-CAM and Grad-CAM + + produce the strongest drop in model confidence when the most relevant pixels are progressively removed. The model’s probability decreases sharply within the first 20–40% of perturbation, indicating that these methods effectively identify critical image regions used by the classifier. The AUPC analysis confirms our choice of Grad-CAM as the optimal XAI method, as it achieves the lowest AUPC value (18.2116), indicating the highest faithfulness in feature attribution.

**Table 8:** AUPC values for saliency maps (lower values indicate higher faithfulness in feature attribution).

XAI method	AUPC value
Grad-CAM	18.2116
Grad-CAM ++	18.461
Score CAM	18.978

### Calibration analysis

To assess the quality of the predictions, we perform a series of tests under calibration analysis of both the baseline and the proposed TrustNet framework. Typically, the calibration measures the correlation between the model’s predicted confidence and the empirical accuracy. The Expected Calibration Error (ECE) quantify the average difference between the predicted confidence intervals and the observed accuracy over probability clusters^70^, Maximum Calibration Error (MCE) reports the worst-case deviation across clusters^70^, and the Brier score is the mean squared error between the probabilities and ground truth labels^71^. In general, lower values indicate better calibration. Furthermore, we applied temperature scaling, a post hoc calibration method in which the logits are divided by a learned scaler temperature to increase the precision of the prediction probabilities. This ensures better alignment in model confidence with observed results without altering classifier decisions^72^. In Table 9, we provide all the results for these metrics.

**Table 9:** Calibration results.

Model	ECE	MCE	Brier	ECE Temp scaled	MCE Temp scaled	Brier Temp scaled	Temp
Baseline	0.110815	0.860979	0.070385	0.138419	0.847868	0.071346	0.789736
Trustnet	0.116625	0.854361	0.074924	0.125981	0.847287	0.070249	1.351179

For all the settings, the brier scores remain low, thus indicating accurate predictions. Temperature scaling improves TrustNet’s Brier score from 0.0749 to 0.0702, indicating enhanced prediction quality. The ECE values show a relatively small mismatch between predicted confidence and accuracy, with temperature scaling improving TrustNet’s ECE to 0.1260 compared to the baseline’s 0.1384. The MCE results are 0.8610 for the baseline and 0.8544 for TrustNet, indicating a greater deviation between confidence and accuracy across probability clusters. This miscalibration is reduced after temperature scaling, indicating that the TrustNet model is reasonably well calibrated.

**Fig. 8:**
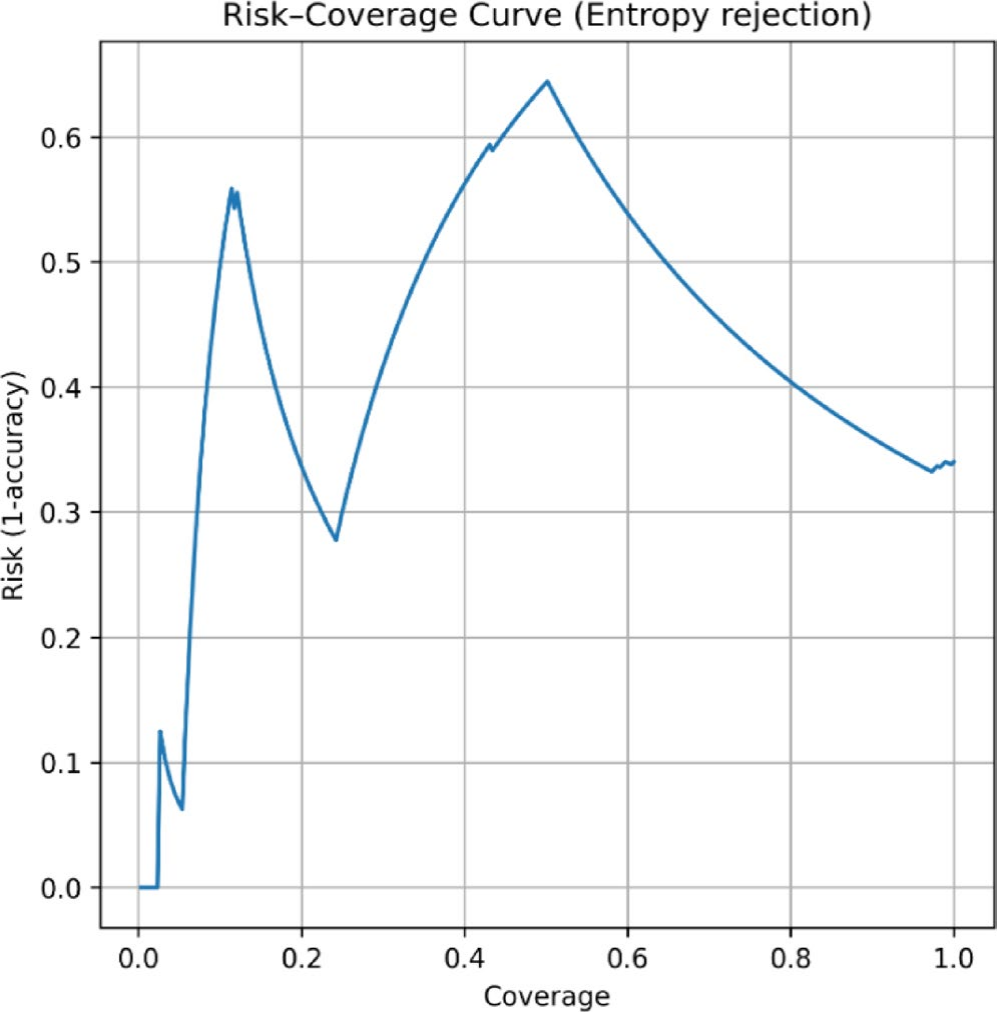
Reliability diagram for TrustNet model.

To further strengthen the utility aspect of the proposed work, we perform risk-coverage analysis^73,74^ on the basis of the entropy rejection criteria, as shown in Fig. 8. In this scenario, models refrain from prediction for high uncertainty cases, and coverage denotes the fraction of samples where the model gives predictions. The risk is computed as 1-accuracy for these samples. Generally, a suitable model must show low risk at high coverage, which implies that it provides reliable prediction for most cases. In our results, the risk-coverage curve shows a clear downwards trend as the rejection threshold increases. At the high coverage portion (where all samples are retained), the risk is naturally high because difficult and confusing cases are included. As coverage decreases, risk decreases substantially, reflecting that discarded cases are indeed those for which the model is uncertain and that the prediction might be wrong. Thus, the model prioritizes confident predictions while refraining from confusing or uncertain cases. The spikes observed may be due to a small set of high-confidence misclassifications, a common occurrence in medical datasets^73–76^, but once these errors are removed, the risk substantially decreases.

## Discussion

Compared with several baselines, the proposed shallow CNN for ischemic stroke classification pertaining to CT imaging exemplifies its compelling performance in multiple folds of evaluation. Integrating UQ (via MCD) and XAI (via Grad-CAM) has resulted in our model being accurate, interpretable, and reliable, the three features indispensable for clinical translation. Further important insight from our research underlines the significance of beyond accurately explaining support. While achieving high accuracy on test sets is crucial for AI technologies, their clinical adoption remains largely limited to research settings. This limitation emphasizes the need to establish both user trust and proper interpretability of results. Our methodology, which integrates the quantitative XAI together with the MCD, meets these requirements by providing confidence scores with visual explanations. This dual approach enables clinicians to understand not only the model’s predictions but also their associated confidence levels and the specific image regions influencing each decision. More importantly, rather than limiting XAI to model development and validation, we apply it during inference to provide additional information and reduce uncertainty in real-time clinical predictions. Our research demonstrates that the addition of the UQ module and then the combined UQ + XAI method has incrementally improved performance. Type I errors (false alarms for ischemic stroke) have been gradually reduced from one configuration to another, thereby providing strong evidence that uncertainty quantification and explainability work together to avoid overconfident and uncertain misclassifications. These results demonstrate that combining these techniques creates a system that enhances safety by accurately identifying suspected cases for expert review, thus reducing the risk of misclassification. The conservative approach of our framework of flagging uncertain cases for expert review, rather than risking harmful misclassifications, enhances clinical safety. This performance highlights the effectiveness of our architecture design, which achieves robust results through residual pathways, dropout regularization, and adaptive pooling techniques, while maintaining lower computational overhead compared to established encoders like ResNet, EfficientNet, and VGG.

### Clinical interpretation using heatmaps

**Fig. 9:**
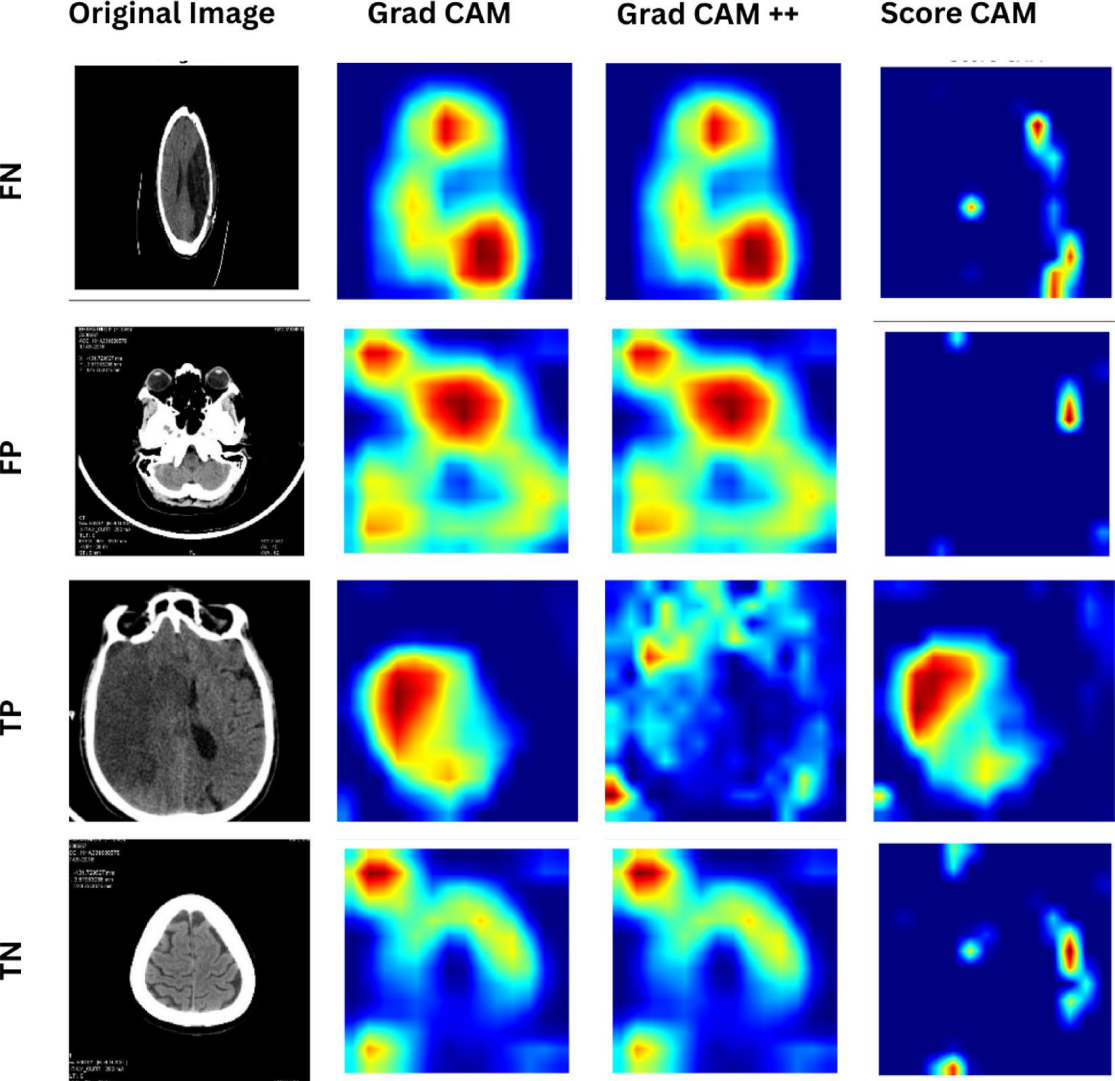
Saliency maps generated by Grad-CAM, Grad-CAM++, and Score-CAM for randomly selected cases showing TP, FN, FP, and TN predictions.

Saliency maps, as utilized in this work, play an important role in making the model’s decision transparent and interpretable. To make this information useful for clinicians, we randomly chose 6 images from our dataset and produced heatmaps through Grad-CAM, Grad-CAM + +^78^ and Score-CAM^79^, as shown in Fig. 9. The interpretation of these maps reveals distinct patterns across different prediction outcomes:

- In TP cases, all methods highlight regions that correspond well with visible infarcts, typically hypodense cortical or deep gray–white matter areas. The heatmaps provide information on clinically relevant areas, indicating that the model’s decision is driven by the actual brain ischemic lesion.
- FN examples usually involve subtle or early ischaemic changes where hypodensity is faint, ambiguous, or partially confounded by normal anatomical variations. The saliency maps show diffuse or misplaced activations, indicating that the model failed to recognize the subtle lesion.
- FP heatmaps reveal activations over nonimportant regions, suggesting that the model incorrectly attributes normal density variations to ischemia. Clinicians recognize these features as normal rather than stroke features, highlighting the need for improved model robustness.
- In TN patients, heatmaps are either minimally activated or highlight nondiagnostic regions, which is consistent with the findings of normal studies. Clinically, this indicates that the model correctly avoids classifying normal anatomical regions as infarcts, supporting its reliability in ruling out stroke.

### Strengths

- *High diagnostic performance*: The method has a maximum accuracy of 98.94%, accompanied by a sensitivity of 100%, implying that no stroke cases are overlooked, which is a requirement in medical environments.
- *XAI-based better transparency*: Grad-CAM integration offers spatial attention maps to reveal the brain areas that matter most for model prediction.
- *UQ integrated*: The model utilizing MCD predicts the confidence of every prediction and marks the doubtful cases for expert review, which avoids overconfidence error and consequently increases the model’s reliability in the clinical decision-making process.
- *Robustness tests with private and public datasets*: The model trained and tested on both private and public datasets of CT scans. .
- *Balanced trade-offs among accuracy, Interpretability, and efficiency*: TrustNet has proven that it is possible to maintain world-class accuracy and, at the same time, does not sacrifice interpretability and computational efficiency.

### Advantages

- *Lightweight architecture*: The use of heavy and old models, such as VGG-16 or ResNet-152, results in high processing power and computational costs, whereas TrustNet is built with only 0.66 million parameters, which is an excellent choice for real-time implementations and edge computing in resource-scarce environments.
- *Transparency extending via XAI*: The combination with Grad-CAM provides spatial attention maps indicating the regions of the brain that are important to the predictions of the model.
- *Integrated UQ*: Involvement of MCD ensures that the system will not only predict the confidence of each case but also that a doubtful case will be marked for the expert’s attention. This would prevent an error of overconfidence, thus extending the reliability of the model in clinical decision-making.
- *Robustness proved on private and public sets*: The model was trained and evaluated on a private dataset as well as on 2023 well-curated annotated CT scan datasets indicating the different stroke phases.
- *Accuracy-interpretability-efficiency trade-off*: TrustNet is an example in which state-of-the-art accuracy can be reached while not sacrificing interpretability and computational efficiency.

### Limitations

- *Binary classification scope*: The scope of the research is to perform binary classification using brain CT images.
- *Clinical metadata*: Key clinical variables such as NIHSS scores were not consistently available in our retrospective single-center dataset; however, we plan to incorporate them in prospective studies to increase their clinical relevance. All the images were annotated by experienced radiologists.
- *Dataset generalizability and UQ approach*: While our model shows promising results, the private dataset’s single-institution nature (94 patients) raises concerns about learning institution-specific features rather than generalizable pathological patterns. Although our strong performance on the external public dataset partially mitigates this concern, future work would benefit from multi-institutional data collection. This would not only ensure broader generalizability but also enable the implementation of more sophisticated uncertainty quantification methods like deep ensembles^77^, which could provide more robust uncertainty estimates than the current MCD approach.

## Conclusion and future work

We propose TrustNet, a lightweight and interpretable CNN for collecting information from CT images to diagnose ischemic stroke. The MCD-based UQ and Grad-CAM methods for XAI have been synergistically combined to provide a more trustworthy and transparent diagnostic framework. The proposed model yields 94.67% and thus 6.17% improvement in accuracy. TrustNet is characterized by a mere 0.66 million parameters and outperforms classical encoders in terms of performance. The use of MCD in uncertainty estimation and Grad-CAM for visual explanation not only makes the classification robust but also makes the predictions transparent and confidence aware. These features ensure that the model is very suitable for real-time implementation in clinical and resource-limited areas. Alongside Grad-CAM, we also developed a novel quantitative metric, which is the mean intensity of the saliency map, that helps to evaluate attention consistency under stochastic inference. To be more precise, the metric was able to distinguish right and wrong predictions, particularly when it was used in conjunction with the Grad-CAM methods. Future research will also involve extending the framework for multiclass stroke stratification, employing multimodal imaging input, and thereafter, prospective clinical validation will be carried out to determine the real-world diagnostic workflow utility. We will also explore adaptive threshold calibration techniques to ensure robustness across different clinical settings. This would be an essential contribution to the research community in instilling confidence and explainability in DL models, aiding radiologists with another layer of confirmation for decision making.

## Data Availability

The datasets generated during and/or analysed during the current study are not publicly available owing to ethical restrictions but are available from the corresponding author upon reasonable request.
